# Prognostic significance of A-kinase interacting protein 1 expression in various cancers: A meta-analysis based on the Chinese population

**DOI:** 10.1097/MD.0000000000029203

**Published:** 2022-06-24

**Authors:** Shanshan Xue, Chuanmeng Zhang, Jie Xu, Chenglin Zhou

**Affiliations:** a Department of Clinical Laboratory, Taizhou People's Hospital, Affiliated 5 to Nantong University, Taizhou, Jiangsu Province, China; b The Center for Translational Medicine, Taizhou People's Hospital, Affiliated 5 to Nantong University, Taizhou, Jiangsu Province, China.

**Keywords:** AKIP1, cancer, meta-analysis, prognosis

## Abstract

**Background:**

Cumulative evidence suggests that A-kinase interacting protein 1 (AKIP1) plays an important role in tumor progression. However, the prognostic value of AKIP1 expression in various cancers remains unclear. Here, we conducted a meta-analysis to evaluate the prognostic value of AKIP1 expression in patients with cancer.

**Methods:**

The PubMed, Web of Science, EMBASE, CNKI, and Wanfang databases were systematically searched to identify studies in which the effect of AKIP1 expression on prognosis (overall survival or disease-free survival) was investigated. Hazard ratios (HRs) with 95% confidence intervals (CIs) were combined to assess the effect of AKIP1 expression on patient survival. Odds ratios (ORs) with 95% CIs were pooled to estimate the association between AKIP1 expression and clinicopathological characteristics of patients with cancer.

**Results:**

Nineteen eligible studies, encompassing 3979 patients, were included in the meta-analysis. AKIP1 expression was negatively associated with overall survival (HR = 1.86, 95% CI: 1.58–2.18, *P* < .001) and disease-free survival (HR = 1.69, 95% CI: 1.53–1.87, *P* < .001) in patients with cancer. Moreover, AKIP1 overexpression was positively correlated with adverse clinicopathological features, such as tumor size (OR = 2.22, 95% CI: 1.67–2.94, *P* < .001), clinical stage (OR = 2.05, 95% CI: 1.45–2.90, *P* < .001), depth of tumor invasion (OR = 2.98, 95% CI: 2.21–4.02, *P* < .001), and degree of lymph node metastasis (OR = 2.12, 95% CI: 1.75–2.57, *P* < .001).

**Conclusions:**

High AKIP1 expression is an unfavorable prognostic biomarker and may serve as a potential therapeutic target in patients with cancer.

## 1 Introduction

In recent years, tumors have become the main cause of death worldwide; they poses a serious health risk and are a major public health problem in terms of both medical and socioeconomic burdens.^[1]^ According to the Global Cancer Observatory report, there were 18.1 million new cancer cases and 9.6 million cancer deaths globally in 2018^[2]^; of these, 48.4% and 57.3%, respectively, occur in Asia.^[2]^ Although great progress has been made in various treatment strategies for cancer, the survival rate of patients with many types of cancers remains unsatisfactory, mainly owing to the malignant progression of tumors.^[3]^ Biomarkers can be used as important tools for tumor diagnosis, as therapeutic targets, and as predictors of clinical outcomes.^[4,5]^ It is imperative to identify reliable prognostic biomarkers for individualized treatment strategies to improve the clinical outcomes of patients with cancer.^[6]^

A-kinase interacting protein (AKIP1), also known as breast cancer-associated protein 3, was originally identified in breast and prostate cancer cell lines via mRNA screening.^[7]^ It is an intracellular protein localized in the cytoplasm, nucleus, and mitochondria, acting as an adaptor of intracellular structural proteins.^[8,9]^ AKIP1 is a binding partner of the p65 subunit in the nuclear factor-kappa B (NF-κB) signaling pathway.^[10]^ It also induces the protein kinase A catalytic subunit to enhance the transcriptional activity of NF-κB via phosphorylation, which promotes the progression of several tumors.^[11,12]^ Furthermore, AKIP1 promotes angiogenesis and tumor growth by elevating the concentrations of NF-κB-dependent chemokine ligand 1, CXCL2, and CXCL8.^[13]^ In addition, AKIP1 promotes epithelial-mesenchymal transformation via Slug-mediated signaling in gastric cancer cells, activation of the PI3K/Akt/IKKβ pathway in cervical cancer cells, and transactivation of zinc finger E-box-binding homeobox 1 in non-small cell lung cancer (NSCLC) cells, promoting cancer cell migration and invasion.^[8,14–16]^ Moreover, AKIP1 is overexpressed in various cancers.^[12,15–23]^ Thus, clinical studies have shown that AKIP1 overexpression is associated with poorer survival in a variety of cancers.^[12,15–21,23–30]^ However, such conclusions remain controversial.^[8,17,22]^ Therefore, we performed a meta-analysis to further explore the relationship of AKIP1 expression with prognosis and clinical characteristics in patients with cancer.

## 2 Materials and methods

This was a systematic review and meta-analysis based on published articles. Thus, no ethical approval was required.

### 2.1 Literature search

This meta-analysis was conducted in accordance with the Preferred Reporting Items for Systematic Reviews and Meta-Analyses.^[31]^ The PubMed, Web of Science, EMBASE, CNKI, and Wanfang databases were comprehensively searched for relevant studies (up to September 3, 2021) in which the association between AKIP1 expression and prognosis (including overall survival [OS] and disease-free survival [DFS]) was evaluated in patients with various cancers. The following terms were used in the search: (“A-kinase interacting protein 1” OR “AKIP1” OR “breast cancer-associated protein 3” OR “BCA3”) AND (“cancer” OR “tumor” OR “carcinoma” OR “malignancy” OR “neoplasm”) AND (“prognosis” OR “survival” OR “outcome”). In addition, references cited in the retrieved articles were manually reviewed to identify eligible studies that may have been overlooked during the database search.

### 2.2 Inclusion and exclusion criteria

Candidate studies were included in the meta-analysis based on the following criteria:

1. the patients were all from the Chinese population and were diagnosed with cancers via pathological or histological examinations;
2. the relationship between the expression of AKIP1 and the prognosis of cancer patients was evaluated;
3. expression of AKIP1 was detected via immunohistochemistry (IHC), quantitative reverse transcription polymerase chain reaction (qRT-PCR), or western blotting (WB);
4. patients were divided into 2 groups based on the expression level of AKIP1; and
5. hazard ratios (HRs) and their 95% confidence intervals (CIs) could be directly extracted from the article or estimated based on sufficient information.

Articles were excluded in accordance with the following criteria:

1. case reports, reviews, letters, or conference abstracts;
2. basic research, or animal experiments;
3. patients were not divided into 2 groups based on AKIP1 expression; and
4. studies without sufficient information to estimate the HR and the corresponding 95% CI.

### 2.3 Data extraction and quality assessment

The following data from eligible studies were extracted by 2 independent researchers: first author's name, publication year, province, cancer type, clinical stage, follow-up time, sample size, detection method, cut-off value, clinicopathological features, OS, DFS, HR and 95% CI, and analytical method. If the article provided HR values for both univariate and multivariate analyses, we chose the latter because of higher accuracy after adjusting for confounding factors. In addition, if an article did not provide an HR value, we estimated it according to the Kaplan–Meier survival curve.

The Newcastle-Ottawa Scale was adopted to evaluate the methodological quality of eligible articles in three dimensions: selection, 0 to 4; comparability, 0 to 2; and outcome, 0 to 3.^[32]^ Studies with an overall score of ≥6 were considered of high quality.

### 2.4 Statistical analysis

HRs and 95% CIs were combined to examine the relationship between AKIP1 expression and the prognosis of patients with cancer. Odds ratios (ORs) and 95% CIs were pooled to evaluate the association between AKIP1 expression and the clinicopathological features. The Chi-Squared test and *I*^2^ statistic were used to test heterogeneity among the studies. *P* < .05 or *I*^2^ > 50% was considered as significant heterogeneity, and then a random-effects model was applied for analysis. Otherwise, a fixed-effects model was used. Sensitivity analysis was conducted via sequential deletion of a single included study to confirm the stability of our results, and funnel plot, Begg test, and Egger test were used to assess potential publication bias. All statistical analyses were conducted using STATA software, version 12.0 (Stata Corporation, College Station, TX), and a two-sided *P* < .05 was considered statistically significant.

## 3 Results

### 3.1 Search results and study characteristics

**Figure 1:**
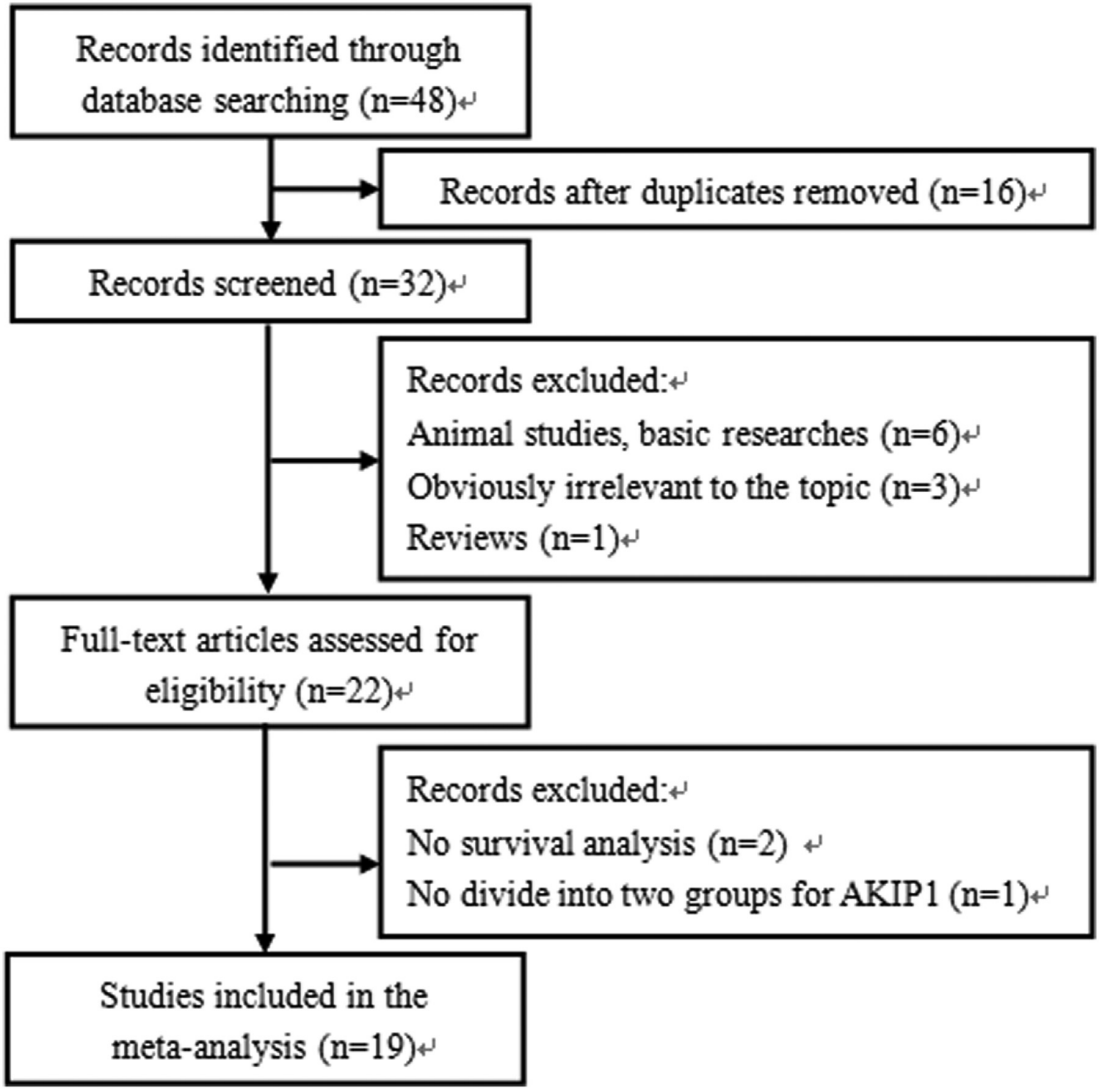
Flowchart presenting the steps of literature search and selection.

The article selection process is illustrated in Figure 1. During our systematic literature search, we identified 48 articles, of which 16 were duplicates. After screening the titles and abstracts, 10 were removed because they were animal studies, basic research, irrelevant articles, or reviews. We assessed the eligibility of the remaining 22 full-text articles. We excluded 2 in which no survival analysis was performed, and one in which the patients were not divided into 2 groups based on the expression level of AKIP1. Finally, 19 studies with 19 cohorts were included in this meta-analysis.

The 19 cohorts, with a total of 3979 patients, were recruited throughout China, and patients were diagnosed with multiple types of cancer, that is tongue squamous cell carcinoma,^[8]^ papillary thyroid carcinoma,^[17]^ acute myeloid leukemia,^[20,28]^ multiple myeloma,^[19]^ prostate cancer,^[25]^ cervical cancer,^[27]^ clear cell renal cell carcinoma,^[11]^ NSCLC,^[16,21,26]^ gastric cancer,^[15,18]^ hepatocellular carcinoma,^[12,24,29]^ colorectal cancer,^[22]^ breast cancer,^[23]^ and epithelial ovarian cancer.^[30]^ The sample sizes ranged from 50 to 490. The expression level of AKIP1 was detected using IHC,^[8,11,12,15–17,21–27,29,30]^ qRT-PCR,^[18,20,28]^ and WB.^[19]^ For all 19 cohorts, the OS was reported,^[8,12,15,11–30]^ and for 9, the DFS was reported.^[12,16,17,21,23,25–27,29]^ According to the Newcastle-Ottawa Scale score, all cohorts were assigned a score greater than or equal to 6, indicating that the articles were of high quality. The detailed characteristics of the included cohorts are described in Table 1.

**Table 1:** Main characteristics of the eligible studies.

Study	Region	Duration	Cancer type	Clinical stage	Follow up (months)	Number	Detection method	Cut-off value	AKIP1-high (%)	Survival analysis	Language	Quality
Sun Y 2021	Hebei	NR	TSCC	I-IV	NR	194	IHC	≥4	109 (56.2)	OS (U)	English	6
Zhang L 2020	Zhejiang	2015–2019	PTC	I-IV	60.0	245	IHC	≥4	144 (58.8)	OS (U), DFS (U)	English	6
Yan Y 2020	Inner Mongolia	2016–2019	AML	M1-M6	Median 18.0	291	qRT-PCR	Median	146 (50.2)	OS (M)	English	8
Wang W 2020	Shanghai	2016–2019	MM	I-III	Median 22.0	152	WB	Median	76 (50.0)	OS (U)	English	6
Wang D 2020	Hubei	2015–2018	PC	NR	Median 27.0	248	IHC	≥4	140 (56.5)	OS (M), DFS (U)	English	8/6
Wan X 2020	Shanghai	2012–2014	CC	I-II	NR	150	IHC	≥3	109 (72.7)	OS (U), DFS (U)	English	6
Peng H 2020	Inner Mongolia	2009–2013	ccRCC	I-III	Median 88.0	210	IHC	≥4	112 (53.3)	OS (M)	English	8
Liu Y 2020	Shandong	2012–2014	NSCLC	I-III	Median 45.5	490	IHC	≥4	263 (53.7)	OS (M), DFS (M)	English	8
Ling J 2020	Guangdong	2013–2018	GC	I-IV	60.0	50	qRT-PCR	>1.35	22 (44.0)	OS (U)	Chinese	6
Fang T 2020	Hubei	2014–2015	HCC	A-B	Median 35.0	432	IHC	≥4	167 (38.7)	OS (M)	English	8
Hao X 2019	Shandong	2016–2019	AML	M1-M6	Median 17.5	160	qRT-PCR	Median	80 (50.0)	OS (U)	English	6
Cui Y 2019	Guangdong	2006–2009	HCC	I-III	NR	223	IHC	NR	117 (52.5)	OS (U), DFS (M)	English	6/8
Chen H 2019	Hebei	2010–2013	NSCLC	I-III	NR	319	IHC	≥4	201 (63.0)	OS (M), DFS (M)	English	8
Chen D 2019	Jiangsu	NR	GC	I-IV	60.0	96	IHC	≥3	62 (64.6)	OS (U)	English	6
Ma D 2018	Henan	2007–2010	HCC	I-III	NR	107	IHC	≥4	54 (50.5)	OS (U), DFS (U)	English	6
Jiang W 2018	NR	NR	CRC	I-IV	60.0+	251	IHC	≥4	139 (55.4)	OS (M)	English	7
Guo X 2017	Henan	2008–2011	NSCLC	I-IV	60.0	139	IHC	≥4	81 (58.3)	OS (U), DFS (U)	English	6
Mo D 2016	Guangxi	1998–2004	BC	I-IV	NR	150	IHC	≥4	69 (46.0)	OS (M), DFS (M)	English	8
Zhang H 2012	Zhejiang	2007–2009	EOC	I-III	NR	72	IHC	≥1	41 (56.9)	OS (U)	Chinese	6

AML = acute myeloid leukemia, BC = breast cancer, CC = cervical cancer, ccRCC = clear cell renal cell carcinoma, CRC = colorectal cancer, DFS = disease-free survival, EOC = epithelial ovarian cancer, GC = gastric cancer, HCC = hepatocellular carcinoma, IHC = immunohistochemistry, M = multivariate analysis, MM = multiple myeloma, NR = none reported, NSCLC = non-small cell lung cancer, OS = overall survival, PC = prostate cancer, PTC = papillary thyroid carcinoma, qRT-PCR = quantitative reverse transcription polymerase chain reaction, TSCC = tongue squamous cell carcinoma, U = univariate analysis, WB = western blot.

### 3.2 Association between AKIP1 expression and clinicopathological features

**Figure 2:**
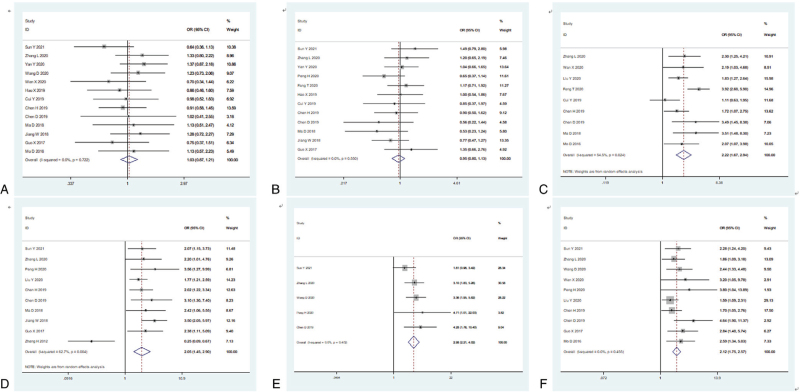
Forest plot reflecting the association between AKIP1 and clinicopathological features in cancer patients. (A) age (young vs old); (B) gender (female vs male); (C) tumor size (small vs large); (D) clinical stage (I-II vs III-IV); (E) depth of invasion (T1-T2 vs T3-T4); (F) lymph node metastasis (negative vs positive).

First, we explored the relationship between AKIP1 expression and clinicopathological features (Table 2, Fig. 2). The results indicated that high expression levels of AKIP1 were positively related to certain phenotypes of tumor aggressiveness, including tumor size (OR = 2.22, 95% CI: 1.67–2.94, *P* < .001, Fig. 2C), clinical stage (OR = 2.05, 95% CI: 1.45–2.90, *P* < .001, Fig. 2D), depth of tumor invasion (OR = 2.98, 95% CI: 2.21–4.02, *P* < .001, Fig. 2E), and degree of lymph node metastasis (OR = 2.12, 95% CI: 1.75–2.57, *P* < .001, Fig. 2F). However, no relationship was observed between AKIP1 expression and age (OR = 1.03, 95% CI: 0.87–1.21, *P* = .743, Fig. 2A) or sex (OR = 0.95, 95% CI: 0.80–1.13, *P* = .569, Fig. 2B).

**Table 2:** Meta-analysis of AKIP1 and clinicopathological features in cancer patients.

Categories	Trials (Patients)	OR (95% CI)	*I*^2^(%)	*P*_*h*_	*Z*	*P*_z_
Age (young vs. old)	13 (2573)	1.03 (0.87–1.21)	0.0	.722	0.33	.743
Gender (female vs. male)	12 (2667)	0.95 (0.80–1.13)	0.0	.550	0.57	.569
Tumor size (small vs. large)	9 (2212)	2.22 (1.67–2.94)^R^	54.5	.024	5.51	<.001
Clinical stage (I-II vs. III-IV)	10 (2123)	2.05 (1.45–2.90)^R^	62.7	.004	4.06	<.001
Depth of invasion (T1-T2 vs. T3-T4)	5 (993)	2.98 (2.21–4.02)	0.0	.472	7.13	<.001
Lymph node metastasis (negative vs. positive)	10 (2241)	2.12 (1.75–2.57)	0.0	.455	7.73	<.001

All pooled ORs were calculated from fixed-effects model except for cells marked with (random^R^). *P*_*h*_ denotes *P* value for heterogeneity based on Q test; *P*_*z*_ denotes *P* value for statistical significance based on Z test. CI = confidence interval, OR = odds ratio.

### 3.3 Association between AKIP1 expression and prognosis

**Figure 3:**
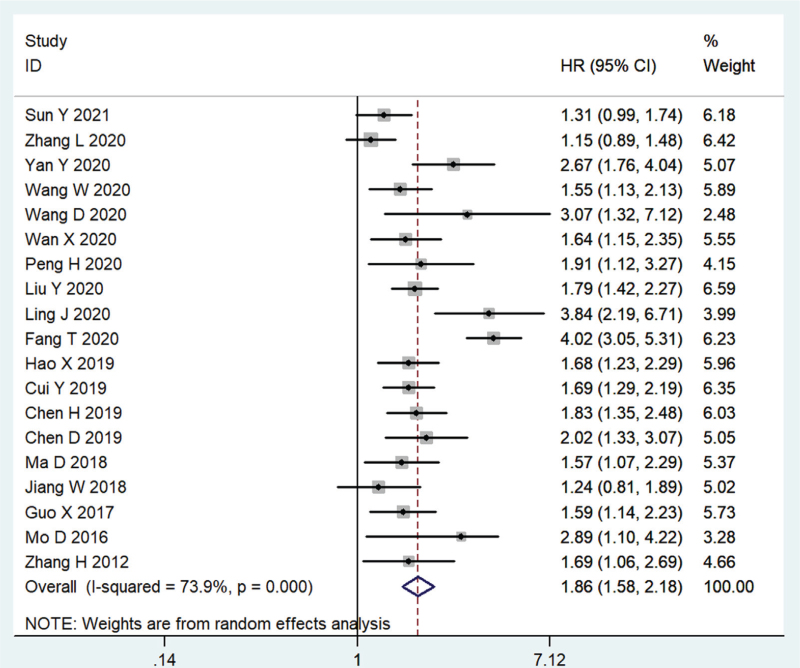
Forest plot illustrating the relationship between AKIP1 expression and overall survival (OS) of cancer patients.

As indicated in Table 3, a cumulative meta-analysis was conducted to evaluate the prognostic value of AKIP1 in human cancers. In terms of the relationship between AKIP1 expression and OS, the studies were heterogeneous (*I*^2^ = 73.9%, *P* < .001); therefore, a random-effects model was used during analysis. Overall, a high expression level of AKIP1 was negatively associated with OS (HR = 1.86, 95% CI: 1.58–2.18, *P* < .001, Fig. 3).

**Table 3:** Summary of the meta-analysis results.

Categories	Trials	HR (95% CI)	*I* ^2^ *(%)*	*P*_*h*_	*Z*	*P*_*z*_
OS (All)	19 (3979)	1.86 (1.58–2.18)	73.9	<.001	7.52	<.001
Cancer type						
Digestive system	6 (1159)	2.15 (1.44–3.20)	86.2	<.001	3.75	<.001
Genitourinary system	4 (680)	1.79 (1.41–2.28)^F^	0.0	.587	4.76	<.001
Blood system	3 (603)	1.86 (1.38–2.50)	55.2	.107	4.07	<.001
HNC	2 (439)	1.22 (1.01–1.47)^F^	0.0	.481	2.03	.042
NSCLC	3 (948)	1.76 (1.49–2.06)^F^	0.0	.803	6.81	<.001
BC	1 (150)	2.89 (1.10–4.22)	–	–	–	.022
Clinical stage						
Stage I-IV	7 (1125)	1.70 (1.28–2.25)	73.3	.001	3.68	<.001
Stage I-III	7 (1573)	1.71 (1.52–1.93)^F^	0.0	.981	8.76	<.001
Stage I-II	1 (150)	1.64 (1.15–2.35)	–	–	2.70	.007
M1-M6	2 (451)	2.07 (1.32–3.25)	67.0	.082	3.18	.001
A-B	1 (432)	4.02 (3.05–5.31)	–	–	9.81	<.001
NR	1 (248)	3.07 (1.32–7.12)	–	–	2.61	.009
Detection method						
IHC	15 (3326)	1.79 (1.49–2.15)	75.4	<.001	6.15	<.001
qRT-PCR	3 (501)	2.47 (1.55–3.95)	73.4	.023	3.79	<.001
WB	1 (152)	1.55 (1.13–2.13)	–	–	2.70	.007
Sample size						
>200	9 (2709)	1.95 (1.46–2.59)	84.6	<.001	4.56	<.001
≤200	10 (1270)	1.68 (1.49–1.89)^F^	40.2	.090	8.60	<.001
Analysis method						
Multivariate	8 (2391)	2.24 (1.66–3.02)	78.0	<.001	5.30	<.001
Univariate	11 (1588)	1.61 (1.40–1.86)	48.1	.037	6.57	<.001
DFS (All)	9 (2071)	1.69 (1.53–1.87)	39.7	.103	10.50	<.001

All pooled HRs were calculated from random-effects model except for cells marked with (fixed^F^). *P*_*h*_: *P* value for heterogeneity based on Q test; *P*_*z*_: *P* value for statistical significance based on Z test. BC = breast cancer, CI = confidence interval, DFS = disease-free survival, HNC = head and neck cancer, HR = hazard ratio, IHC = immunohistochemistry, NR = none reported, NSCLC = non-small cell lung cancer, OS = overall survival, qRT-PCR = quantitative reverse transcription polymerase chain reaction, WB = western blot.

We attempted to better clarify the prognostic value of AKIP1 expression for OS by conducting subgroup analyses, according to cancer type, clinical stage, detection method, sample size, and analytical method (Table 3). Subgroup analysis according to cancer type indicated that increased AKIP1 expression was closely associated with an unfavorable OS in patients with digestive system cancers (HR = 2.15, 95% CI: 1.44–3.20, *P* < .001), genitourinary system cancers (HR = 1.79, 95% CI: 1.41–2.28, *P* < .001), blood system cancers (HR = 1.86, 95% CI: 1.38–2.50, *P* < .001), head and neck cancers (HR = 1.22, 95% CI: 1.01–1.47, *P* = .042), NSCLC (HR = 1.76, 95% CI: 1.49–2.06, *P* < 0.001), and breast cancer (HR = 2.89, 95% CI: 1.10–4.22, *P* = .022). Stratified analysis according to clinical stage revealed that elevated AKIP1 expression reduced OS in patients with stage I-IV (HR = 1.70, 95% CI: 1.28–2.25, *P* < .001), stage I-III (HR = 1.71, 95% CI: 1.52–1.93, *P* < .001), stage I-II (HR = 1.64, 95% CI: 1.15–2.35, *P* = .007), M1-M6 (HR = 2.07, 95% CI: 1.32–3.25, *P* = .001), A–B (HR = 4.02, 95% CI: 3.05–5.31, *P* < .001), and none reported (HR = 3.07, 95% CI: 1.32–7.12, *P* = .009) cancer. In addition, subgroup analysis according to the detection method revealed that high AKIP1 expression was associated with poor OS when determined via IHC (HR = 1.79, 95% CI: 1.49–2.15, *P* < .001), qRT-PCR (HR = 2.47, 95% CI: 1.55–3.95, *P* < .001), and WB (HR = 1.55, 95% CI: 1.13–2.13, *P* = .007). The relationship between high AKIP1 expression and poor OS was statistically significant for sample size >200 (HR = 1.95, 95% CI: 1.46–2.59, *P* < .001), sample size ≤200 (HR = 1.68, 95% CI: 1.49–1.89, *P* < .001), multivariable analysis (HR = 2.24, 95% CI: 1.66–3.02, *P* < .001), and univariate analysis (HR = 1.61, 95% CI: 1.40–1.86, *P* < .001). Thus, a negative correlation was observed between AKIP1 expression and OS in all the subgroups.

**Figure 4:**
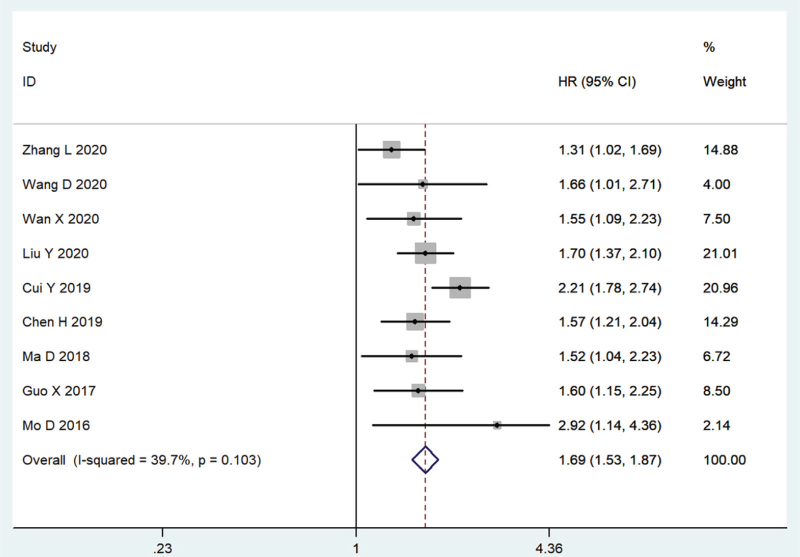
Forest plot illustrating the relationship between AKIP1 expression and disease-free survival (DFS) of cancer patients.

For the nine cohorts (2071 patients) in which the DFS was provided, the pooled results revealed that a high AKIP1 expression was associated with a poor DFS (HR = 1.69, 95% CI: 1.53–1.87, *P* < .001, Fig. 4), and there was no heterogeneity among the cohorts (*I*^2^ = 39.7%, *P* = .103).

### 3.4 Sensitivity analysis and publication bias

**Figure 5:**
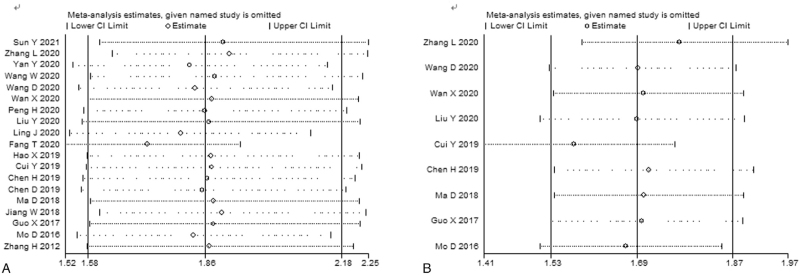
Sensitivity analyses of studies regarding overall survival (A) and disease-free survival (B).

**Figure 6:**
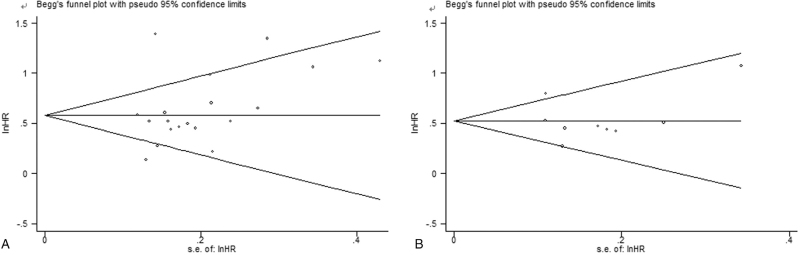
Begg funnel plots for overall survival (A) and disease-free survival (B).

In order to explore the stability of this meta-analysis, sensitivity analysis was performed by omitting single cohort in turn to recalculate the pooled results. The combined HR estimates for OS (Fig. 5A) and DFS (Fig. 5B) were not changed significantly, indicating that our results were stable. In addition, there was no potential publication bias for OS and DFS, which was confirmed by Begg test (OS: *P* = .080; DFS: *P* = .754), Egger's test (OS: *P* = .227; DFS: *P* = .945) and funnel plot (OS: Fig. 6A; DFS: Fig. 6B).

## 4 Discussion

Although the prognostic value of AKIP1 has been studied in various cancers it remains controversial. To our knowledge, this was the first meta-analysis in which the prognostic value of AKIP1 expression was evaluated in patients with various cancers. Our study indicated that high AKIP1 expression was negatively associated with OS and DFS in patients with cancer. Sensitivity analysis and publication bias analysis indicated that the results were stable and reliable. Subgroup analysis demonstrated that high AKIP1 expression was a predictor of poor OS, independent of cancer type, clinical stage, detection method, sample size, and analytical method. In addition, we evaluated the relationship between AKIP1 expression and clinicopathological features. The results revealed that high AKIP1 expression was positively correlated with tumor size, clinical stage, depth of tumor invasion, and degree of lymph node metastasis.

Although our meta-analysis yielded strong evidence, several limitations should be considered when the results are interpreted. First, all cohorts in this study were from China, which limited the generalizability of the results. Second, multiple detection methods and inconsistent cut-off values for AKIP1 expression were used in the included studies, which may have led to the high degree of heterogeneity for the association between AKIP1 expression and OS. Finally, several HRs and 95% CIs were obtained from univariate analyses or estimated from the Kaplan-Meier survival curve, rather than being directly obtained from multivariable analysis, which may have led to bias.

In summary, AKIP1 expression was negatively associated with prognosis and positively associated with adverse clinicopathological features. AKIP1 expression may prove to be an effective prognostic marker, and AKIP1 may be a promising target for treatment of patients with cancer.

## Author contributions

**Conceptualization:** Chenglin Zhou

**Data curation:** Jie Xu, Shanshan Xue

**Funding acquisition:** Chenglin Zhou

**Investigation:** Jie Xu, Shanshan Xue

**Methodology:** Chuanmeng Zhang

**Software:** Chuanmeng Zhang

**Supervision:** Chenglin Zhou

**Writing – original draft:** Chuanmeng Zhang, Shanshan Xue

**Writing – review & editing:** Chenglin Zhou
